# Diagnostic Dilemma Regarding Postpartum Seizure in the Setting of Preeclampsia

**DOI:** 10.7759/cureus.57832

**Published:** 2024-04-08

**Authors:** Jeevan Jangam

**Affiliations:** 1 Department of Obstetrics and Gynecology, Ipswich Hospital, Brisbane, AUS; 2 School of Medicine, Griffith University, Gold Coast, AUS

**Keywords:** preeclampsia, obstetrics and gynecology, immigrant, epilepsy by ncc, nonepileptic seizures

## Abstract

This case demonstrates a diagnostic dilemma regarding the management of seizures in the postnatal setting. It seeks to highlight the importance of a thorough exploration of history and risk factors for females presenting with seizures in the postpartum period to ensure appropriate treatment and workup. Preeclampsia (PET) is a hypertensive disorder of pregnancy affecting 2%-8% of pregnancies worldwide. Less than 1% of females with preeclampsia experience eclamptic seizures. However, they are associated with significant mortality and morbidity. The majority of these seizures occur in the third trimester with 80% occurring intrapartum or within 48 hours of delivery. Warning symptoms such as headache, visual disturbances, or epigastric pain are not always present. Eclamptic seizures after the first week postpartum are very rare. Seizures, in general, are triggered by many other organic and nonorganic causes, one of which is neurocysticercosis (NCC) most often caused by *Taenia solium*. Neurocysticercosis is endemic in sub-Saharan Africa and Southeast Asia. In endemic areas, it accounts for approximately 30% of the cases of adult-onset epilepsy, second only to tuberculosis (TB).

This is a case of a 40-year-old para 1 female who experienced a postpartum seizure in the setting of preeclampsia diagnosed in the intrapartum period. She successfully underwent standard treatment for the management of eclampsia. Further workup demonstrated brain lesions suspicious for neurocysticercosis, ultimately prompting diagnosis and appropriate neurological management. Neurocysticercosis is a commonly overlooked etiology in the Australian peripartum healthcare setting. It must be included in the differential diagnosis of patients with new-onset seizures who may be from endemic areas. The diagnosis of neurocysticercosis is based on a combination of clinical findings, exposure history, imaging, and serology.

## Introduction

Adult-onset seizures need special attention as they can be due to an identifiable cause. The pregnancy period complicates these scenarios due to the physiologic and pathologic processes of pregnancy. Seizures postpartum are often associated with eclampsia. Preeclampsia (PET) can evolve into eclampsia, affecting 0.1% of pregnancies in Australia [1]. Eclamptic seizures are associated with significant mortality and morbidity. Not all females will have warning symptoms such as headache, visual disturbances, or epigastric pain [1]. Eclamptic seizures after the first week postpartum are very rare. A broad differential for seizures should be considered the further they occur from delivery. Outside of eclampsia, seizures occur mainly due to trauma, central nervous system infections, space-occupying lesions, cerebrovascular accidents, metabolic disorders, and drugs [2]. Cysticercosis is the most common parasitic disease of the nervous system in humans and the single most common cause of acquired epileptic seizures in the developing world. It is caused by the larval stage of the tapeworm *Taenia solium* [3]. This case report presents a case of a diagnostic dilemma in managing a postpartum seizure in a female with preeclampsia.

## Case presentation

A 40-year-old primiparous female of Congolese heritage re-presented on day 14 following an emergency cesarean section with an episode of tonic-clonic seizure in the community. Her pregnancy was complicated by risk factors of age, class 1 obesity, partum hemorrhage, and intrapartum PET diagnosis managed with captopril 50 mg twice a day postpartum.

She initially presented to the delivery suite of a busy outer metropolitan hospital in Australia in spontaneous labor at 40 weeks and three days of gestation in September 2023. She underwent an emergency lower-segment cesarean section due to fetal distress in advanced labor and delivered a healthy 3.6 kg baby with an appearance, pulse, grimace, activity, and respiration (APGAR) score of 9 at one and five minutes. Her blood pressures were labile in the postpartum period for 48 hours, reaching a maximum of 170/98 mmHg when assessed manually on the upper arm with the standard adult-size cuff. Preeclampsia was diagnosed due to hypertension in conjunction with an elevated urine protein-creatinine ratio of 32. Her blood pressure readings in the peripartum interval were grossly deranged compared to her prenatal and early pregnancy blood pressure readings, which ranged from 110 to 125 mmHg systolic and 52 to 72 mmHg diastolic. She has no other medical or surgical history. She reported compliance with captopril and denied any recreational substance use. Of note, she migrated from Zambia in 2022.

The seizure was terminated with intramuscular and intravenous midazolam given by the paramedics. She was also administered a 4 mg loading dose of magnesium sulfate. This was followed by a 24-hour infusion during her admission. Her Glasgow Coma Scale (GCS) score of 7 improved rapidly upon presentation. Investigations revealed normal hemoglobin, platelets, white cell count, electrolytes, and biochemistry.

Given the timeframe from delivery, the obstetric team had a high index of suspicion for alternate causes and requested a medical admission. A detailed history revealed an experience of water scarcity and occasional consumption of dirty water. She denied a history of tuberculosis (TB), malaria, or cholera. She reported consuming pork products but did not suspect undercooked or spoilt pork. She did however report that seizures were experienced by some of her community members in Zambia.

**Figure 1:**
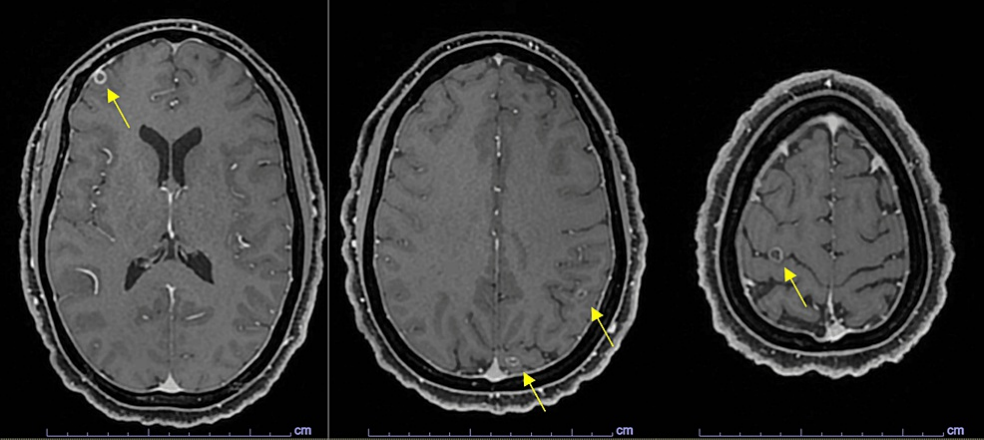
MR of the brain sequence of the patient demonstrating neurocysticercosis. Note the numerous cystic lesions with perilesional edema, particularly at the grey-white junction.

Serology for TB, neurocysticercosis (NCC), and HIV returned negative for all infections and infestations. The imaging of the brain was conducted with CT venogram that demonstrated multiple calcifications in the brain parenchyma. The MR of the brain as an outpatient delineated these lesions as ring-enhancing lesions within the supratentorial grey-white matter interface and periventricular regions, some of which demonstrated perilesional edema, consistent with NCC lesions in the colloidal vesicular and granular nodular phase. See Figure 1.

She was commenced on 2 g of levetiracetam loading dose for seizure prophylaxis as an inpatient. Following a normal EEG and neurology appointment, the levetiracetam was ceased. She was advised to undergo surveillance by way of imaging as she has been stable postpartum and to consider further treatment if there was evidence of progressive disease on imaging or worsening symptomatology. At the three-month postdelivery review in the outpatient clinic, the patient was well and seizure-free. At this appointment, the events leading up to delivery and the readmission were discussed. Risks carried into the next pregnancy were also discussed as the patient reports not yet completing her family.

## Discussion

NCC, most often caused by *Taenia solium*, is a major cause of adult-onset seizures, second only to TB [2,3]. It is endemic in developing countries, particularly in sub-Saharan Africa and Southeast Asia [4]. It is associated with nearly one-third of seizure disorders in endemic areas, and an estimated 50 million people worldwide have the infection [4]. Seizures in the postpartum setting are naturally suspicious to be secondary to obstetric causes such as preeclampsia. However, a detailed history and timing of the seizure event in relation to delivery suggest a low index of suspicion for an obstetric cause [5,6]. In the Australian healthcare setting, cases such as these do not present frequently, leading to a delay in diagnosis and care.

*Taenia solium* is a two-host zoonotic cestode. In its adult stage, it is a 2-4 m-long tapeworm that lives in the small intestine of humans. No other final hosts are known for *T. solium* tapeworms in nature. Humans are usually exposed to eggs by the ingestion of food (often undercooked or spoilt pork) or water contaminated with feces containing these eggs or proglottids [3,4]. Tapeworm carriers can also infect themselves through fecal-oral transmission (e.g., caused by poor hand hygiene). Once eggs or proglottids are ingested, oncospheres hatch in the intestine, invade the intestinal wall, enter the bloodstream, and migrate to multiple tissues and organs where they mature into cysticerci over 60-70 days [4]. Some cysticerci will migrate to the central nervous system, causing neurocysticercosis (NCC). The pathophysiology of the perilesional edema surrounding these neurocysticercoses is not certain. These lesions are suspected to be the cause of epileptiform activity leading to adult-onset epilepsy [2-4]. Patients often present with nonspecific symptoms such as headaches or seizures. Neuroimaging, CT or MRI, is the gold standard for diagnosing NCC. This may be further aided by serology and fecal cultures; however, they may not be positive as sensitive and specific tools for diagnosing NCC or taeniasis are lacking [4].

Routine anti-epileptic medications such as benzodiazepines and levetiracetam are used to manage seizure activity. Corticosteroids address neuronal edema, and anti-parasitic medications such as praziquantel and albendazole are the mainstays of treatment. In severe cases, neurosurgical input may be sought to remove epileptiform foci [3]. The impact of pregnancy on NCC and consequently seizures is not well known. There is a paucity of literature to determine if pregnancy is associated with an increased risk of the reactivation of NCC. It is thought that immune-suppressed states may trigger intermittent release or the recognition of parasite antigens by the host and a possible inflammatory response [5]. Furthermore, while pregnancy is not known to increase the frequency in those females with seizures, the physiologic changes of pregnancy can decrease the efficacy of anti-epileptic medications [7]. There is limited literature regarding the effects of preeclampsia on epilepsy. Rat studies have demonstrated up to 44% reduction in the seizure threshold in epileptic rats in the context of preeclampsia [8]. This limitation in literature opens an avenue for further research.

## Conclusions

The paucity of reported cases prevents us from assessing whether there is an increased risk of NCC reactivation in pregnancy. In this case, the seizure was determined to be likely due to NCC in the context of a reduced seizure threshold due to PET. Diagnosing NCC is a challenging process as patients may be entirely asymptomatic or present with nonspecific symptoms such as seizures or headaches. Pregnancy and, in particular, preeclampsia further complicate accurate diagnosis and management of this finding. In settings of large immigrant populations, obstetricians and gynecologists may encounter patients with NCC. Taking a thorough history and assessment of individualized risk factors will facilitate appropriate and safe management.
